# Investigation of alveolar osteitis and the effectiveness of laser treatment: a unified Meta-analysis and review of the literature

**DOI:** 10.1186/s12903-024-04461-w

**Published:** 2024-06-17

**Authors:** Alessio Rosa, Alberto Maria Pujia, Claudio Arcuri

**Affiliations:** 1 Department of Chemical Science and Technologies, Dentistry, University of Tor Vergata, Rome, 00133 Italy; 2 Department of Biomedicine and Prevention, University of Rome Tor Vergata, Rome, 00133 Italy; 3 Department of Clinical Sciences and Translational Medicine, University of Rome Tor Vergata, Rome, 00133 Italy

**Keywords:** Dry socket, Laser therapy, Alveolar osteitis

## Abstract

**Background:**

Post-tooth extraction, dry socket is a frequently encountered complication, causing substantial pain and hindering the healing process. Conventional approaches to manage this condition have traditionally involved the use of antiseptic dressings to diminish bacterial presence and facilitate healing. This study aims to assess the efficacy of laser therapy in the symptomatic treatment of alveolitis.

**Methods:**

A literature search was conducted on PubMed, Embase, Scopus, Google Scholar, Web of Science, focusing on publications from 1998 to 31/01/2024 using relevant keywords. The combination of “laser” and “dry socket” was executed through the boolean connection AND.

**Results:**

At the conclusion of the study, a total of 50 studies were identified across the three search engines, with only three selected for the current systematic study and meta-analysis. The meta-analysis indicated that laser treatment proves effective in addressing alveolitis compared to Alvogyl. However, the correlation between the two was not highly significant.

**Conclusion:**

These findings suggest that laser therapy may serve as a viable alternative to traditional treatments for dry socket. This minimally invasive procedure has the potential to alleviate pain and promote healing with fewer associated side effects.”

## Introduction

Dry socket frequently arises following tooth extraction, causing intense pain and hindering the healing process [1]. Traditional treatments for this condition have relied on antiseptic dressings to mitigate bacteria and promote healing. Recent research suggests that laser therapy, a minimally invasive procedure, could serve as an effective alternative to conventional dry socket treatments [1].

Tooth extraction is a common dental procedure that can lead to the postoperative complication known as dry socket, occurring when the prematurely lost blood clot after extraction results in severe pain, delayed healing, and infection [1, 2]. Conventional treatments involve antiseptic dressings, such as those with chlorhexidine and eugenol, with some disagreement on the ideal therapeutic protocol. Laser treatment has emerged as a recent alternative, utilizing low-level lasers to reduce inflammation, enhance wound healing, and alleviate pain in a relatively painless, single-session procedure [2]. 

A study comparing laser treatment and antiseptic dressings in treating dry socket included 60 patients who underwent tooth extraction. The laser treatment group experienced significantly less pain, faster healing, and fewer side effects compared to the antiseptic dressing group. Low-level laser therapy (LLLT) has gained recognition for its positive impact on inflammatory processes, wound healing, and antimicrobial effects when applied to oral mucosa [3]. 

The SaliCept patch, a freeze-dried acemannan hydrogel preparation, represents another recent treatment option. Acemannan, derived from Aloe vera L., is the main component with known antimicrobial properties. This review aims to assess the effects of laser therapy on post-extraction alveolitis, a common complication of surgical extractions [4]. 

## Materials and methods

### Criteria for inclusion and exclusion

We assessed the eligibility of each document based on the Population, Exposure, Comparator, and Outcomes (PECO) model, considering the following parameters:

(P) Participants: Individuals who have undergone extraction and are experiencing dry socket. (E) Exposure: Patients with alveolar osteitis (AO) undergoing laser therapy. (C) Comparison: Patients with AO undergoing various forms of therapy. (O) Outcome: The primary objective is to evaluate the efficacy of laser treatment in patients with AO, with a secondary focus on its preventive effectiveness.

Inclusion Criteria:

1. Articles written in English.
2. Clinical trials with laser treatment.
3. Randomized clinical trials with laser treatment.

Exclusion Criteria:

1. Non-PECO articles.
2. Duplicate articles.
3. Books.
4. Letters to editors and experimental studies.
5. Non-English language studies.
6. Studies involving animals.
7. Radiochemotherapy patients.
8. Review articles.
9. Case series.
10. Case reports.
11. Patients with systemic diseases.

### Search methodology

To identify relevant publications, we systematically searched the PubMed, Web of Science, and Lilacs databases for articles published from January 1998 to 31/01/2024. Additionally, manual searches were conducted for systematic reviews on the same topic from previous periods. The following scientific sources were analysed: Pubmed, Scopus, Web of Science, Google Scholar, Embase. MeSH phrases were applied in PubMed, while manual searches compensated for their absence in other search engines (Table 1).

**Table 1:** Search strategy

**PubMed**
(“dry socket“[MeSH Terms] OR (“dry“[All Fields] AND “socket“[All Fields]) OR “dry socket“[All Fields]) AND (“laser s“[All Fields] OR “lasers“[MeSH Terms] OR “lasers“[All Fields] OR “laser“[All Fields] OR “lasered“[All Fields] OR “lasering“[All Fields])
***Web of Science***
((ALL=(dry socket)) AND ((ALL=(laser))
***Google scholar***
“dry socket” AND “laser”

### Study characteristics

Upon concluding the study and utilizing the three search engines, a total of 50 studies were identified. At the initial phase, 13 items were excluded due to duplication, and an additional 5 were disqualified based on language barriers. During the initial screening, 27 articles were eliminated as they were systematic reviews, not meeting the inclusion criteria. A specific filter was applied to include only randomized clinical trials. The final screening involved assessing the abstracts of five publications.

**Fig. 1:**
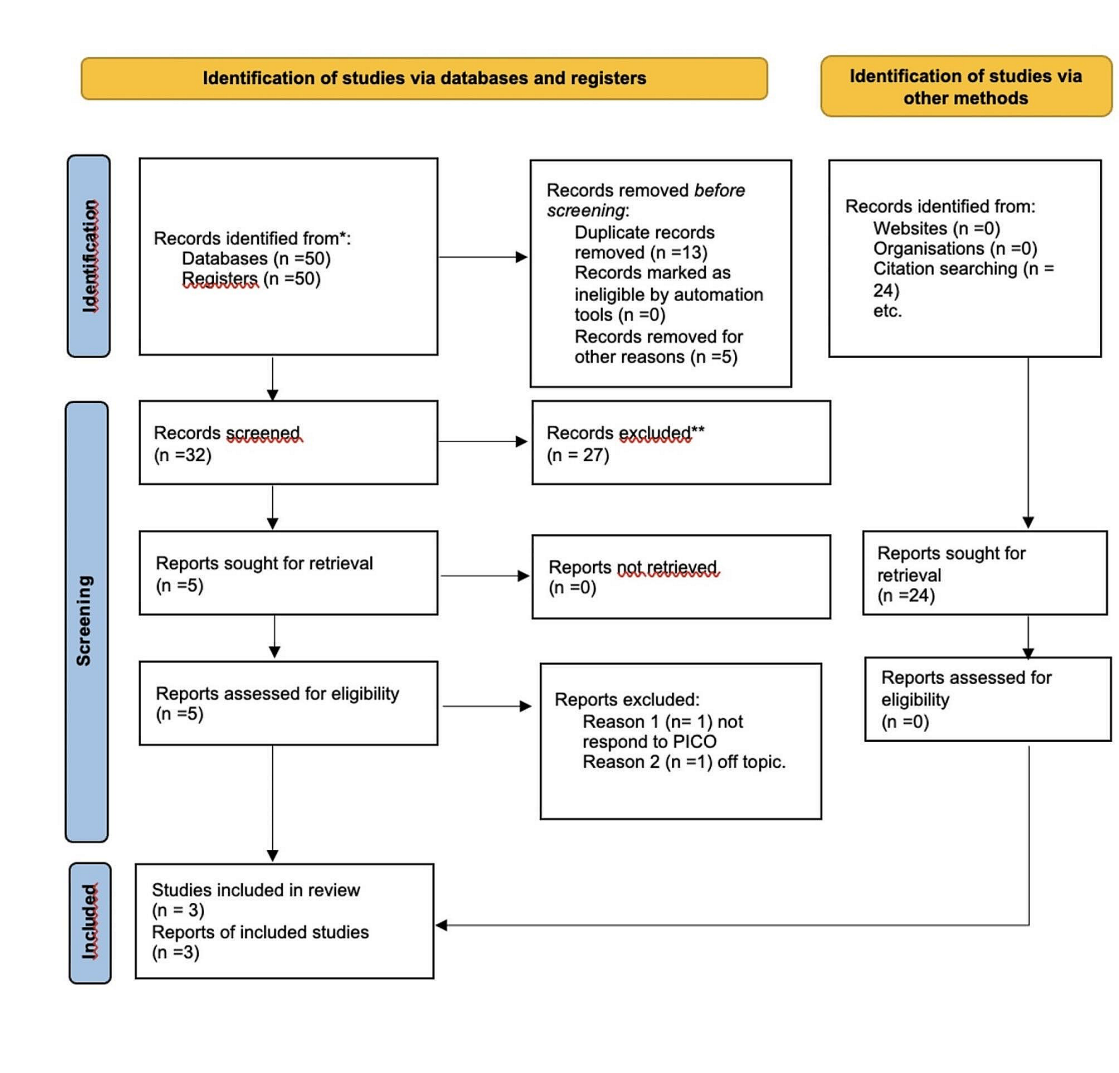
Prisma flowchart

The creation of the present systematic study adhered to the PRISMA 2020 flowchart (Fig. 1), resulting in the selection of three studies. Two articles were excluded: one did not align with the PECO criteria, and another addressed AO incidence without a control group. Papers were initially screened based on the PECO model, and three articles were identified through search engines. A manual search from bibliographies and websites was also conducted, leading to the selection of 24 articles. However, after reviewing their abstracts, some were excluded for not meeting the PECO criteria or being duplicates.

The selected studies in this meta-analysis span from 2009 to 2024. Abstracts were thoroughly read, and groups comparing Alvogyl and laser were considered, while those involving mechanical therapy were excluded from the meta-analysis. Notably, the studies exhibit heterogeneity as they do not uniformly consider laser power and setting. All studies utilized the VAS scale to assess post-alveolitis pain. The research was conducted across different regions globally, including Arabia, Iran, and the USA, involving a total of 119 subjects. Among them, 60 were in the study group evaluating laser efficacy, and 59 were in the control group treated with Alvogyl. All studies followed a randomized controlled trial (RCT) design, ensuring two or more groups. Pain scale assessments were consistently conducted after 3 days across all studies to maintain study homogeneity.

### Data Retrieval

Two reviewers (A.R. and A.P.) independently collected information from the included papers using a customized data extraction process on an Excel spreadsheet. In case of disagreements, a third reviewer (C.A.) facilitated reaching a consensus. Extracted information included: (1) First author; (2) Year of publication; (3) Sample; (4) Therapy Type; (5) Pain Assessment; and (6) Therapy Results. Table 2 now incorporates the extracted and added data. Two authors independently read all publications, and the data were cross-referenced and appropriately placed in the table.

**Table 2:** Main characteristics of the studies included in the present systematic review

Author	Year	Sample	Type of therapy	Evaluation of pain	Results of therapy
ALharti et al.	2023	55 Patients: Group 1: 14 Group 2: 13 Group 3: 14 Group 4: 14	Group 1: MC Group 2: MC with Alvogyl Group 3: Alvogyl with PBMT Group 4: PBMT	VAS scale	Alvogyl is more efficient in the treatment of pain
Eshghpour et al.	2015	60 Patients: Group 1:20 Group 2: 20 Group 3: 20	Group 1: Alvogyl Group 2: LPRL Group 3: LPIL	VAS scale	Low laser therapy has a good effect to treat AO
Kaya et al.	2011	104 Patients: Group 1: 26 Group 2: 26 Group 3: 26 Group 4: 26	Group 1: courettage Group 2: MC with Alvogyl Group 3: MC with salicept Group 4: MC with LLT	VAS scale	Low-level laser therapy treatment at 7.64 J/cm2 (0.1 W 60 s 6 J) performed superiorly in managing alveolar osteitis

### Statistical analysis

Pooled analyses were conducted using Review Manager version 5.2.8 (Cochrane Collaboration, Copenhagen, Denmark; 2014). Alvogyl therapies combined with curettage were compared to laser therapy in treating alveolar osteitis (AO). Inverse variance with random effects was employed for comparing different therapies [5]. The Risk ratio between the two groups was measured. Study heterogeneity was assessed using the Higgins Index (I2) and the chi-square test, categorized as follows: low heterogeneity (< 30%), medium heterogeneity (30–60%), and high heterogeneity (> 60%).

### Quality assessment and risk of bias

**Fig. 2:**
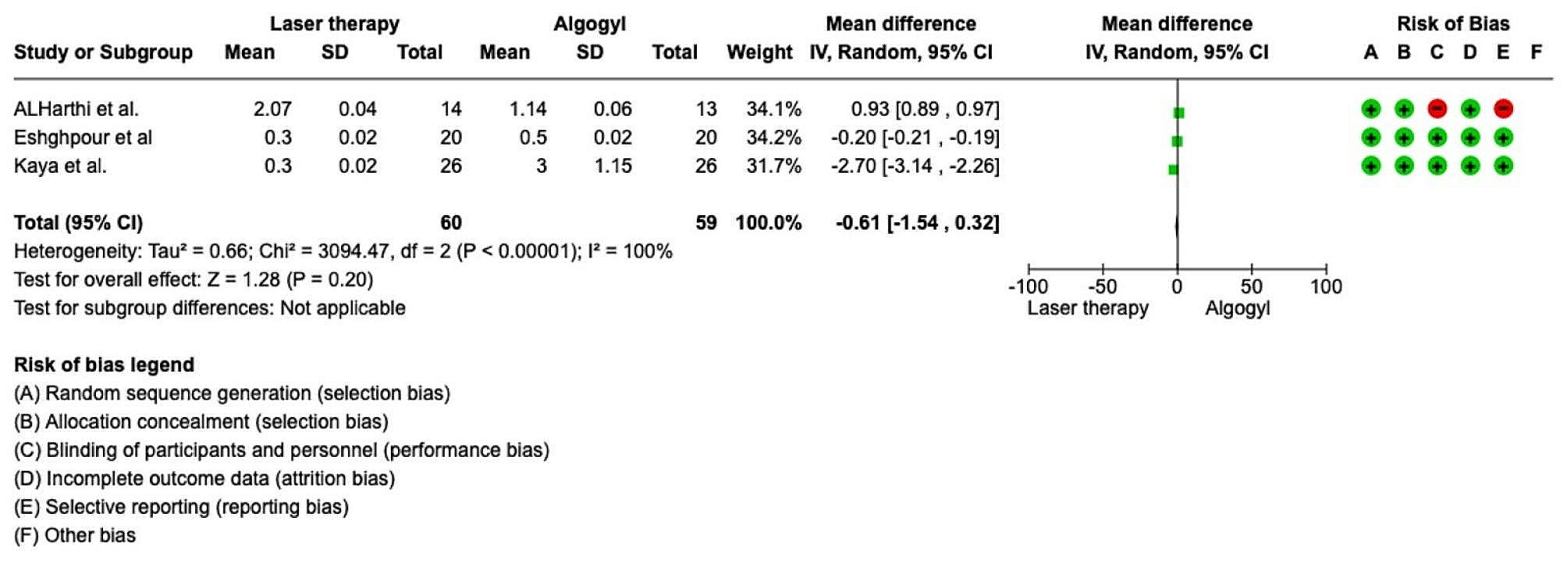
Risk-of-bias domains of included studies

RoB 2 was used to determine the risk of bias, which is shown in Fig. 2. All of the studies ensured a minimal risk of bias with regard to the randomization process. However, bias in the choice of reported outcomes was adequately removed in 100% of the included research, but only in 75% of the studies for self-reported outcomes. Though 100% of the studies reported complete outcome data, 75% of them eliminated performance bias. Overall, it was found that all 3 investigations had a low likelihood of bias.

## Results

### Main findings

The AlHarthi study [6] focuses on evaluating the pain outcomes using different treatments for alveolar osteitis, commonly known as dry socket. This study is particularly noteworthy as it is the first to assess the combined effect of Alvogyl and photobiomodulation therapy (PBMT) on post-operative pain. Participants were divided into four groups: the first received basic mechanical curettage with saline, the second added Alvogyl dressings to the curettage, the third combined Alvogyl with diode laser therapy, and the fourth used PBMT alone. The study found that the group using Alvogyl with diode laser reported lower pain scores compared to those using Alvogyl alone or PBMT alone, especially on the second and third days post-operation. In Kaya’s study [3], the effectiveness of different treatments for alveolar osteitis was compared among 104 patients. These treatments included curettage and irrigation alone, the combination of curettage with Alvogyl, the application of a SaliCept patch, and diode laser treatment. The results indicated that the diode laser treatment group experienced better outcomes in managing symptoms of alveolar osteitis compared to the other groups, particularly those using Alvogyl or the SaliCept patch. Lastly, Eshghpour’s research [7] examined the effectiveness of low-level laser therapy (LLLT) in the form of red and infrared lasers compared to Alvogyl in treating alveolar osteitis. The study involved three groups: one received Alvogyl after socket irrigation, the second was treated with low-power red laser, and the third with low-power infrared laser. The findings highlighted that initially, the Alvogyl group experienced significantly less pain than the laser groups. However, over time, the red laser group showed a more significant reduction in pain, particularly on the second and third days.

### Metanalysis

**Fig. 3:**
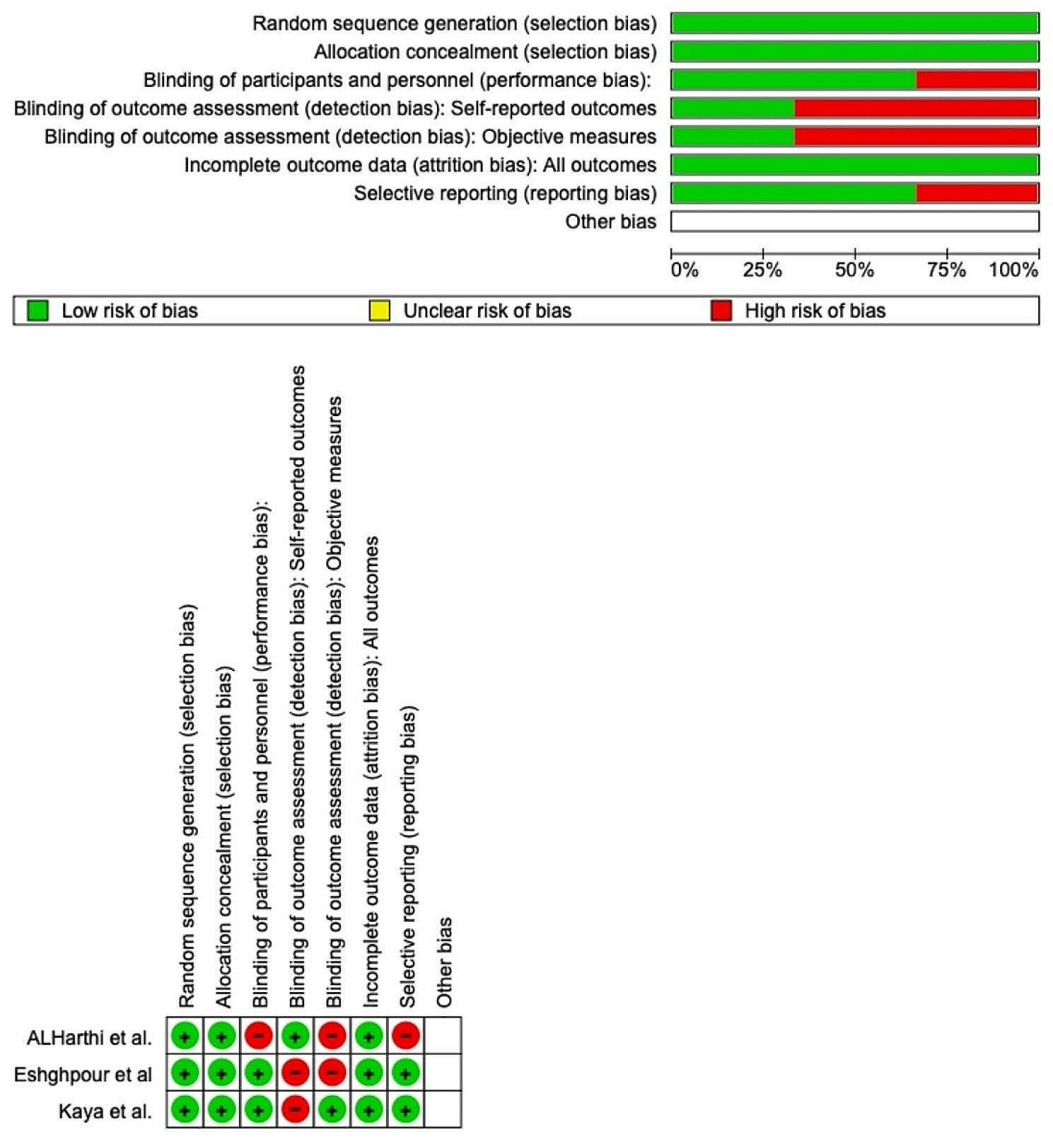
Forest plot of the meta-analysis

The meta-analysis was conducted by random model effect because of the high heterogeneity (*I*2 = 100%) between the 3 included studies [8–10]. The overall effect, reported in the forest plot (Fig. 3), the Forrest plot found that laser therapy has a higher efficacy on the third day on pain (Mean difference − 0.61; CI 95% from − 1.54 to 0.32) with a *p*-value < 0.05.

## Discussion

This review compared various treatments for alveolitis, focusing on three studies that employed Alvogyl and laser application for alveolitis treatment. However, the meta-analysis revealed high heterogeneity, rendering the results unreliable. To enhance homogeneity, the analysis focused on pain assessment using the VAS scale three days after treatment application [3, 11].

Dry socket, affecting up to 10% of post-extraction patients, is associated with severe postsurgical pain. Apart from causing pain, alveolar osteitis (AO) can induce negative psychological impacts, including financial burdens, dental anxiety, and fear of future dental procedures [12, 13]. Alvogyl aids in AO healing, and the current study hypothesized that combining Alvogyl with PBMT is more effective in reducing self-rated post-operative pain than other treatments. Studies suggest that PBMT triggers biochemical mechanisms promoting tissue healing, increases the expression of analgesic facilitator prostatic acid phosphatase, and potentially reduces pain awareness [14, 15]. 

Healing in AO treatment is palliative, typically occurring within 1 to 4 weeks postoperatively. Regardless of the technique used, cleaning and irrigating the extraction socket to remove debris and germs from the denuded bone is crucial [6]. Even patients receiving only curettage and irrigation showed symptomatic improvement, emphasizing the significance of this procedure. Dressing the extraction socket with materials like Alvogyl [3] prevents debris buildup, relieves pain, accelerates healing, and prevents odor, highlighting its importance. Dressing ingredients, such as eugenol in Alvogyl, may contribute to anti-inflammatory and analgesic effects [16, 17]. 

Pain management in AO has utilized LLLT, which may help reduce inflammation and enhance wound healing. However, clinical evidence supporting the superiority of one technique over another is lacking. A study by Kaya et al. found acemannan to be a successful palliative treatment for AO [3], but LLLT led to faster VAS score reductions post-treatment [3, 18]. 

Eshghpour’s study [7] examined three groups. The Alvogyl group experienced reduced pain, while the 660 nm laser group showed consistent pain reduction, and the 810 nm diode laser group demonstrated effective pain reduction over three days. Alvogyl appeared to reduce pain more quickly than LLLT, but the 660 nm laser eventually surpassed Alvogyl initial advantage [3, 7, 19, 20]. 

The healing process was accelerated by both red and infrared lasers [3]. PBM Therapy significantly decreased the risk of developing AO in the first postoperative week, indicating its preventive potential. While Alvogyl may provide quick relief for post-alveolitis pain, laser treatment emerges as a helpful and user-friendly option for both pain prevention and treatment [3, 21–23]. 

In conclusion, the findings suggest that laser treatment could be an effective alternative to conventional dry socket treatments, offering minimally invasive relief from pain and promoting healing with fewer side effects. However, further research is needed to assess the long-term efficacy of laser treatment for drysocket [20, 24, 25].

## Data Availability

The data from the present study can be obtained upon reasonable request from the corresponding author.

## Declarations

### Disclaimer

The statements, opinions and data contained in all publications are solely those of the individual author(s) and contributor(s) and not of MDPI and/or the editor(s). MDPI and/or the editor(s) disclaim responsibility for any injury to people or property resulting from any ideas, methods, instructions or products referred to in the content.
